# A new version of the grapevine reference genome assembly (12X.v2) and of its annotation (VCost.v3)

**DOI:** 10.1016/j.gdata.2017.09.002

**Published:** 2017-09-18

**Authors:** A. Canaguier, J. Grimplet, G. Di Gaspero, S. Scalabrin, E. Duchêne, N. Choisne, N. Mohellibi, C. Guichard, S. Rombauts, I. Le Clainche, A. Bérard, A. Chauveau, R. Bounon, C. Rustenholz, M. Morgante, M.-C. Le Paslier, D. Brunel, A.-F. Adam-Blondon

**Affiliations:** a UMR GV, INRA, UEVE, ERL CNRS, 2 rue Gaston Crémieux, 91000 Evry, France; b EPGV US 1279, INRA, CEA, IG-CNG, Université Paris-Saclay, 91000 Evry, France; c Instituto de Ciencias de la Vid y del Vino (CSIC, Universidad de La Rioja, Gobierno de La Rioja), Logroño 26007, Spain; d IGA, via J. Linussio 51, 33100 Udine, Italy; e SVQV, UMR 1131, INRA, Université de Strasbourg, 28 rue de Herrlisheim, 68000 Colmar, France; f URGI, UR 1164, INRA, Université Paris-Saclay, route de Saint-Cyr, 78026 Versailles, France; g IPS2, UMR 1403, INRA, Université Paris-Saclay, Rue de Noetzlin, bât. 630, 91190 Gif-sur-Yvette, France; h Ghent University, Department of Plant Biotechnology and Bioinformatics, Technologiepark 927, 9052 Ghent, Belgium; i VIB Center for Plant Systems Biology, Technologiepark 927, 9052 Ghent, Belgium

**Keywords:** *Vitis vinifera*, Genome, Chromosomes assembly, Gene annotation

**Table:** 

Specifications	
Organism/cell line/tissue	*Vitis vinifera* cv. PN40024
Sex	Hermaphrodite
Sequencer or array type	The scaffold sequences were obtained by whole genome sequencing using the Sanger technology on ABI3730xl sequencers (Applied BioSystems) according to the supplementary information of Jaillon et al., Nature, 2007, 449: 463–468, doi: http://dx.doi.org/10.1038/nature06148. Genotype data were obtained from the GrapeReSeq 20K *Vitis* genotyping chip (https://urgi.versailles.inra.fr/Species/Vitis/GrapeReSeq_Illumina_20K) following the Infinium HD Assay Ultra Protocol (Ilumina Inc.). The *V. vinifera* cv. Kishmish vatkana mate pair sequences were produced using an Illumina HiSeq 2500 sequencer (Illumina Inc.).
Data format	Analyzed
Experimental factors	Three mapping populations were used: • 120 individuals derived from two reciprocal crosses between *V. vinifera* cv. Riesling cl.49 and *V. vinifera* cv. Gewürztraminer cl.643 (Ri × Gw) • 358 individuals derived from a cross between *V. vinifera* cv. Chardonnay and *Vitis* spp. ‘Bianca’ (Ch × Bi) • 192 individuals derived from two reciprocal crosses between *V. vinifera* cv. Syrah and *V. vinifera* cv. Grenache (Sy × Gr)
Experimental features	Grapevine reference genome assembly and annotation *V. vinifera* cv. Kishmish vatkana was used for the generation of mate pair sequences.
Consent	Creative commons non copy left (cc-by): the data can be freely re-used at the condition to cite its authors
Sample source location	The Ri × Gw and the Sy × Gr populations were maintained in experimental units of the Institut National de la Recherche Agronomique (INRA), respectively the Service Experimentation Agricole et Viticole (Colmar, France) and the Domaine de Vassal (Marseillan-Plage, France). The Ch × Bi population and the *V. vinifera* cv. Kishmish vatkana variety (VIVC no. 6277) were maintained in the germplasm collection of the University of Udine at the Experimental Farm A. Servadei (Udine, Italy).

## 1 Direct link to deposited data

http://doi.org/10.15454/1.4962347083032307E12.

http://doi.org/10.15454/1.5009072354498936E12.

## 2 Introduction

The grapevine reference genome was published by Jaillon et al. [1]. The sequence for the first version of the genome, called the 8X version, was obtained using a whole genome shotgun strategy and the Sanger sequencing technology and was assembled from reads representing 8X coverage. Soon after, the assembly was improved through the addition of 4X of additional coverage, including more Bacterial Artificial Chromosome end sequences that greatly improved the scaffolding of the sequence contigs [2], [3]. The corresponding scaffolds and raw sequences were deposited in European Molecular Biology Laboratory (EMBL) archives (FN594950-FN597014, 2065 entries, release 102). A new chromosome assembly was also developed, based on an improved version of the maps used for the 8X genome version [2], [3], [4], [5] and was also archived at EMBL (FN597015-FN597047, 33 entries, release 102): it is referenced in the grapevine community as the 12X.v0 version of the grapevine reference genome. The chromosome sequence scaffolding of this version still necessitated improvements as around 9% of the sequence was not anchored to chromosomes (with the corresponding scaffolds stacked in the “Unknown” chromosome) and 3.5% of the sequence could be assigned to a chromosome but without certain placement and orientation within the chromosome (stacked in additional “random” chromosomes). The chromosome assembly of the grapevine reference genome was therefore further improved using two strategies. First, six parental maps were saturated with SNP markers developed with different strategies. Second, a collection of mate paired sequences generated from 2 kb DNA fragments of *V. vinifera* cv. Kishmish vatkana was used for further scaffolding. This allowed producing the 12X.v2 version of the grapevine genome assembly presented here.

All these versions of the genome assembly have been accompanied by an automatic gene annotation. The annotation for the original 8X genome release included 30,434 genes predicted with the GAZE software [6]. For the 12X genome assembly, two versions of the annotation were distributed with the 12X.v0 release: the v0 version of the annotation was obtained with the GAZE software and the v1 version (CRIBIv1, 29,971 genes) was the result of the union of v0 and a gene prediction performed with the JIGSAW software [7]. Later, an update of the CRIBIv1, focused on the discovery of the splicing variants, was published by the same group [8]. Finally, National Center for Biotechnology Information (NCBI) Refseq released its own version of the gene prediction (27,043 putative genes) as for most of the species with published genomes. The NCBI Refseq was produced with the Gnomon-NCBI eukaryotic gene prediction tool [9]. For the 12X.v2 version of the genome assembly, an annotation was performed in the frame of the European Cooperation in Science and Technology project FA1106 (VCost) using the EUGENE software [10] and generating 33,568 genes. The design of this latter version was under the supervision of the Super-Nomenclature Committee for Grape Gene Annotation of the International Grapevine Genome Program (IGGP, www.vitaceae.org) fitting its recommendation for the gene nomenclature. The annotation initiatives by families that fitted these recommendations were integrated dynamically to the VCost annotation by curating their respective gene models when needed. So far, the following gene families were integrated to this annotation: the terpenoid synthase gene family [11], the stilbene synthases [12], the MADS box [13], the GRAS [14] and the MYB [15] transcription factors families. Here we describe the generation of the VCost.v3 version of the 12X.v2 version of the grapevine genome assembly, based on a comparison and merging of the NCBI-Refseq, VCost and CRIBIv1 annotations and a semi-manual curation and following the recommendations of the IGGP.

## 3 Materials and methods

### 3.1 Plant material

Three mapping populations were used to develop high density genetic maps: (i) a population of 120 individuals derived from two reciprocal crosses between *V. vinifera* cv. Riesling cl.49 and *V. vinifera* cv. Gewürztraminer cl.643 (Ri × Gw) and maintained at the experimental unit Service Experimentation Agricole et Viticole of the Institut National de la Recherche Agronomique (INRA, Colmar, France), (ii) a population of 358 individuals derived from a cross between *V. vinifera* cv. Chardonnay and *Vitis* spp. ‘Bianca’ (Ch × Bi) and obtained at Experimental Farm A. Servadei of the University of Udine but no longer maintained, (iii) a population of 192 individuals derived from two reciprocal crosses between *V. vinifera* cv. Syrah and *V. vinifera* cv. Grenache (Sy × Gr) maintained at the experimental unit Domaine de Vassal (INRA, Marseillan-Plage, France).

### 3.2 Genotyping the Ch × Bi, Sy × Gr and Gw × Ri populations

The development of a first version of the Ch × Bi and Sy × Gr parental maps is described in Cipriani et al. [4] and Canaguier et al. [5]. Possible errors in segregation data were carefully manually reviewed in these maps and their subsequent revised versions [dataset] [16] were used to generate the chromosome assembly presented in this data paper.

For the Gw × Ri maps, total DNA was extracted with Qiagen DNeasy Plant Maxi Kit (Qiagen, Hilden, Germany), according to the manufacturer's instructions except that 1% of polyvinylpyrrolidone (PVP 40,000) and 1% of β-mercaptoethanol were added to the AP1 buffer. DNA was quantified with Quant-it Picogreen dsDNA Assay Kits (InVitrogen, Life Technologies). The samples were normalized at 50 ng/μl in 96-well plates. Genotype data were obtained from the GrapeReSeq 20K Vitis genotyping chip (https://urgi.versailles.inra.fr/Species/Vitis/GrapeReSeq_Illumina_20K) following the Infinium HD Assay Ultra Protocol (Ilumina Inc., San Diego, CA, USA). Data were analyzed using the Genotyping Module V1.9.4 of Illumina's Genome Studio® software (Illumina Inc., San Diego, CA, USA). After genotyping quality check and automatic clustering the SNP allele callings were manually inspected and edited and the parental maps were generated from the data using the R/qtl software [17].

### 3.3 Mate pair sequencing and alignment on the scaffolds of the grapevine genome assembly

Illumina mate-pair reads were produced using circularization by Cre-Lox recombination. The LoxP circularization linker was removed and used to classify reads with DeLoxer [18]. Illumina adapter was removed using Cutadapt [19]. Quality trimming and contaminant removal was performed with erne-filter [20]. Reads with highly duplicated kmers were removed using Kmercounter (http://sourceforge.net/projects/kmercounter/). Reads were aligned to the repeat masked reference genome using the software bowtie2 [21]. Reads not aligning at scaffold ends (max 5000 bp from the ends), with mapping quality lower than 20, or XM, XO and XG flags above, respectively 2, 1 and 4 were discarded with internally developed Perl scripts. Finally, alignments on scaffolds connected by multiple mate-pairs were visually inspected to discard further false positive alignments. Mate pairs were deposited in the NCBI Short Read Archive under the accession number SRR5712111.

### 3.4 Assembly of the chromosomes

Chromosome assembly was achieved in three steps. First, all markers were aligned on the scaffolds of the 12X genome assembly (FN594950-FN597014, EMBL release 102) by Blat [22] and ePCR [23] according to Jaillon et al. [1]. A first ordering was generated based on these results and taking into account only the parental maps. Then, junctions between adjacent scaffolds were confirmed using mate pair information. Only the scaffolds with multiple evidence of correct ordering (anchoring by at least two maps or at least one map and a mate pair junction) were retained in the assembly. Mate pair information was also used for orienting scaffolds. Finally, all the scaffolds tentatively placed at the extremities of the chromosomes were manually inspected for the presence of telomere repeats. This allowed also confirming the anchoring of these scaffold and sometimes to correct or confirm their orientation.

### 3.5 Development of the VCost.v3 version of the *Vitis* genome annotation

#### 3.5.1 Dataset collection

The CRIBIv1, the NCBI Refseq (NCBI *Vitis vinifera* Annotation Release 101: https://www.ncbi.nlm.nih.gov/genome/annotation_euk/Vitis_vinifera/101/) and the VCost annotation were collected. CRIBI v1 and Refseq were developed on the grapevine genome 12X.v0 while the VCost version was developed already on the 12X.v2 using the EUGENE software. In addition, the gene models predicted by GAZE software in the 8X assembly and by ESTs, used by Grimplet et al. [24], but absent from the CRIBI v1 annotation were used for validation of the models but were not considered in the final VCost.v3 annotation because they correspond to truncated, non-functional genes. The CRIBIv1 gene track includes 29,971 gene models, the Refseq one 27,043 gene models and the VCost one 33,568 models. Algorithm and method for annotations were described in Thibaud-Nissen [25] for Refseq, Foissac et al. [10] for the VCost and in Vitulo et al. [8] for the CRIBIv1.

Manually expert-based curated gene families were also mapped on the 12X.v2 genome version: the terpenoid synthases [11], the stilbene synthases and chalcone synthase [12], the MADS box [13], the GRAS [14] and the MYB [15] transcription factors.

#### 3.5.2 Remapping of genes on the grapevine genome V2

**Fig. 1:**
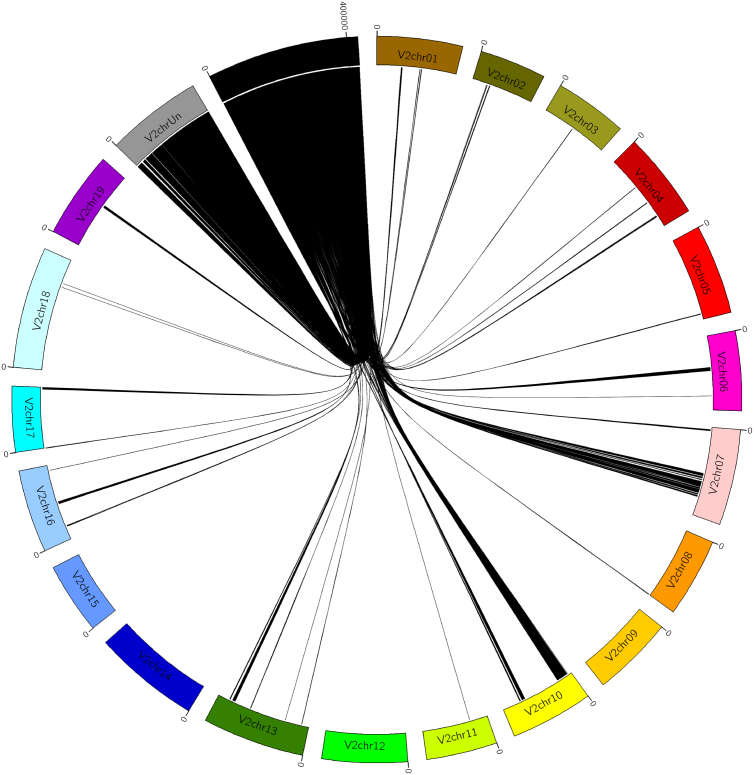
Circular diagram of the transposition of the scaffolds from the unknown chromosome of the 12X.v0 genome assembly (black) to the chromosomes in the 12X.v2 assembly.

CRIBIv1 and Refseq automatic annotations and the expert-based curated gene models were all transposed from genome sequence V0 to V2 using a homemade python script (free source code available at https://github.com/timflutre/VitisOmics/blob/master/src/transferAnnot_from_Vitis_12X_V0_to_V2.pl): since the 12X.v2 assembly was an improvement of the ordering of the scaffolds already used in the 12X.v0 assembly [5], the positions of the features could be deduced from the new position of the scaffolds on the V2 chromosomes (Fig. 1). A JBrowse (http://jbrowse.org/, version 1.11.5) was set up to visualize and give access to these results (https://urgi.versailles.inra.fr/jbrowse/gmod_jbrowse/?data=myData/Vitis/data_gff).

#### 3.5.3 Comparison of annotations and definition of a unique set of gene models

The position of the gene models from the three annotations was compared with a homemade Perl script and overlapping models were grouped together for further analysis.

For each gene, a Blast search was performed against plant protein sequences of the UniProt database except sequences from the *Vitis* genus to avoid self-matching. The 30 best hits with an e-value lower that 1e-20 were kept for further analysis. Two indicators of quality were collected for each gene model: (i) the number of alignments showing an overlapping region of the subject (hit) sequence > 90% (hit overlap value: HO) and (ii) the number of alignments where the overlapping region of the query was > 90% (Query Overlap value: QO). High values of both HO and QO means that the exact structure of the grapevine gene model is frequently found in other species and is likely valid. If the HO number is low and the QO is high, a part of the correct sequence is probably missing in the annotation. If the QO is low and the HO is high, the gene models or known genes from the other plants do not fully cover the grapevine gene model, which may indicate a chimera in the annotation. When both values are low, or in case that there is no hit, the homology only occurs at best on portions of the gene models (subject and query) and keeping the grapevine gene model in the final annotation is questionable. It is important to note that the grapevine coding sequences might not have the same size than in other species but if high HO and high QO were observed for a grapevine gene model from an annotation, this model was preferred over alternative models with lower HO/QO value for inclusion in the final annotation.

If a gene model was only predicted in a single annotation, the locus was added to the final gene set with no further discriminative analysis. If a gene model was predicted by two of the three annotations, the one with the highest HO and QO (> 90%) was chosen in the final set. When a gene model showed equivalent HO and QO scores in more than one annotation, the CRIBI V1 was favored over the VCost that was favored over the Refseq annotation. The main reason to do so, was that the CRIBI V1 was the most widely used version of annotation by the grapevine community, in particular in many published transcriptomic studies. The expert-based manually curated gene models were kept in preference to all the automatic annotations.

#### 3.5.4 Specific case of split or merged gene models

Gene prediction methods can produce inaccurate models resulting in wrong split or merged versions of the actual genes. When such an error occurs in one annotation and not in the others, several genes from each annotation will belong to the same group. These groups were carefully visually inspected with the support of the IGV program [26] to visualize the gene structures from all the annotations. The sequence likely to be correct was conserved. If interpretation was still conflictive, shorter, possibly incomplete structures were favored over longer, possible chimeric, structures.

#### 3.5.5 Construction of the final set of gene models of the Vcost.v3 annotation

Features from conserved gene models for each of the three annotation sets were extracted from their respective initial GFF file and merged into one single GFF file. Feature structure from the three automatic annotations and the six manually curated gene families were standardized and a Locus ID was allocated to each gene following the recommendations of Grimplet et al. [27]. Finally, a file containing both the new sequence and the V3 annotation was prepared at the GenBank sequence format [dataset] [28].

## 4 Results

### 4.1 Development of six parental genetic maps

Six parental maps were developed using three segregating populations, Ri × Gw, Sy × Gr and Ch × Bi, and 2664 non redundant loci. The markers used were SSR markers [4], SNP markers developed from Sanger re-sequencing [5] and for the Ri × Gw progeny, 1580 SNP markers from the 20K grapevine chip. The distribution of the different type of markers in the different maps is described in Table 1.

**Table 1:** Number of loci from the different categories of markers in the six parental maps.

Map	Gw	Ri	Sy	Gr	Ch	Bi
SSR	117	128	288	283	450	466
SNP	750	831	152	94	40	59
Total	867	959	440	377	490	525

**Fig. 2:**
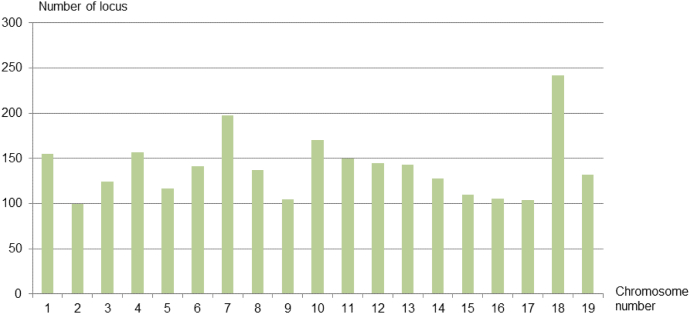
Number of non-redundant loci mapped on each grapevine chromosome using the three segregating populations.

The mapped loci were quite well distributed across the chromosomes: from a 100 loci for the less covered (chromosome 2) to 242 loci for the most covered (chromosome 18; Fig. 2).

The common markers between the maps mainly corresponded to SSR markers (Table 2). These common markers were particularly important to obtain the relative order the contigs anchored in each individual parental maps.

**Table 2:** Number of common loci in each pair of parental maps.

	Gr	Ch	Bi	Ri	Gw
Sy	245	154	154	60	55
Gr		150	148	73	56
Ch			318	83	76
Bi				70	64
Ri					84

The maps and description of the markers are available at [dataset] [16].

### 4.2 Development of the 12X.v2 chromosome assembly

The 2664 non redundant markers were aligned on the scaffolds of the *V. vinifera* reference genome sequence, resulting in a first draft assembly of the chromosomes. A total of 103,463,614 Illumina 100-bp reads were generated from 51,731,807 inserts of average 2 kb size from a single library of *V. vinifera* cv. Kishmish vatkana. These reads were aligned on the scaffolds sequence extremities of the *V. vinifera* reference genome sequence in order to generate links between scaffolds. The alignments were manually inspected, taking into account the data obtained from the genetic maps and resulting in the selection of 2031 mate pairs that joined adjacent scaffolds.

**Fig. 3:**
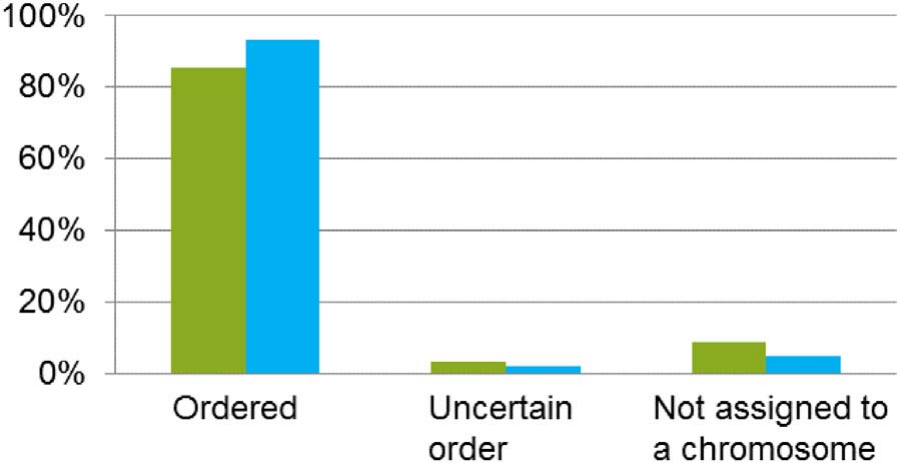
Percentage of the genome sequence (i) ordered on the 19 grapevine chromosomes in the current version of the assembly (12X.v0, in green) and in the new version (12X.v2, in blue), (ii) assigned to a chromosome but with uncertain order or (iii) not assigned to any chromosome.

**Fig. 4:**
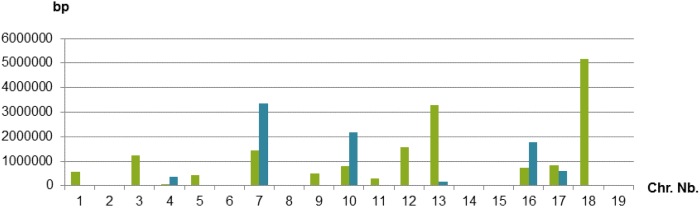
Total size of the sequence scaffolds which order is uncertain for the 19 chromosomes in the 12X.v0 (green bars) compared to the 12X.v2 (blue bars) versions of the grapevine reference genome sequence.

The combination of these two layers of information together with a manual check of the presence of telomeric repeats at the extremity of the chromosomes allowed developing the 12X.V2 chromosome assembly [dataset] [16]. It consists of 19 grapevine chromosomes containing 366 scaffolds totaling 458,641,822 bp. An additional 2,654,308 bp pseudomolecule, named chr00, consists of the remaining 1692 unanchored scaffolds. Compared to the previous version, 8% of unassigned genome sequence is ordered along grapevine chromosomes in the resulting V2 assembly (Fig. 3), although there is still a small portion of the scaffolds which is ordered with some degree of uncertainty, especially on chromosomes 7, 10 and 16 (Fig. 4).

The International Grapevine Genome Program consortium decided to insert these scaffolds at their most likely intra-chromosomal location instead of generating a chrX random pseudomolecule, as we did in the v0 version of the chromosomes assembly. The v2 chromosome assembly therefore consists of 19 chromosome sequences (chr01 to chr19) and one chromosome random pseudo-molecule (chr00). The AGP (Assembly Golden Path) of the chromosomes and the level of uncertainties are described in details in [dataset] [16].

**Fig. 5:**
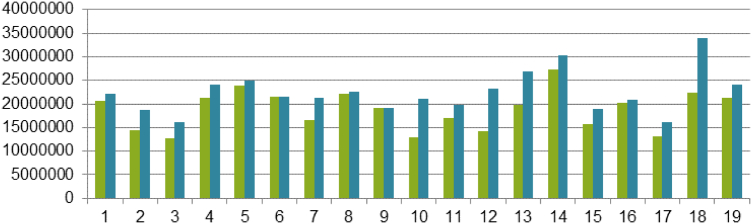
Total size of the scaffolds which are ordered and oriented for each of the 19 chromosomes in the 12X.v0 version of the grapevine genome assembly (green bars) compared to the 12X.v2 version (blue bars).

The 12X.v2 assembly contains more oriented sequence than the 12X.v0 (+14%) and nearly all chromosome sequences benefit from this improvement (Fig. 5). The pair-mate approach contributed importantly to the improvement of the orientation of the scaffolds in the new assembly, confirming the orientation of 75 scaffolds (156.8 Mb) and allowing the orientation of 90 scaffolds (5.3 Mb). This improvement was especially important in regions covered by many small scaffolds.

### 4.3 Development of the VCost.v3 version of the grapevine reference genome annotation

**Fig. 6:**
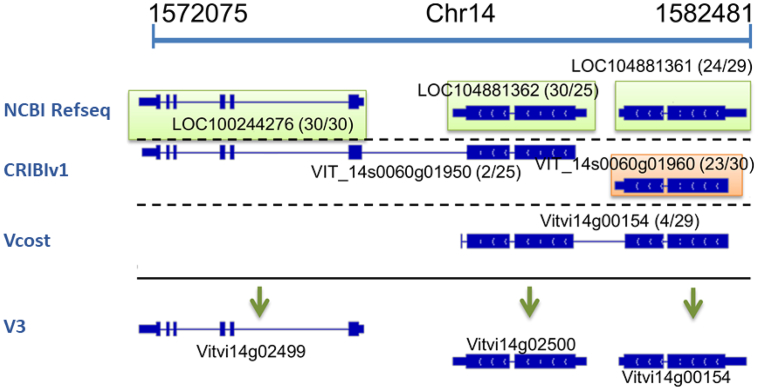
Example of alignment between the gene models from the 3 annotations showing chimeric genes for pectinesterases genes. In brackets HO/QO scores.

An initial blast comparison between the three sets of gene models proposed by the three gene annotations generated 5761 groups containing multiple genes from each of the annotations. The structure of each group was very specific and it was not possible to define an automatic procedure to properly identify the correct gene models within each group. In order to standardize the selection criterion, we defined indicators for each gene taking into account the occurrence of similar gene model in public database based on alignment with proteins from other plant species: the HO and QO described in the material and methods. As an example, Fig. 6 represent a group of adjacent pectinesterase that has been concatenated into chimeras in some annotations.

We observed that the 3 gene models from Refseq (LOC100244276 (30/30), LOC104881362 (30/25), LOC104881361 (24/29)) and one gene model from the CRIBIv1 (VIT14s0060g01960 (23/30)) showing high HO/QO scores whereas the VIT14s0060g01950 (2/25) and Vitvi14g00154 (4/29) models from the VCost did not, for both there are few genes in other species that fully overlap the *Vitis* sequence. These two gene models were likely chimeras from 2 artificially assembled coding sequences corresponding to the Refseq gene models. Besides, predicted proteins for LOC104881362 VIT14s0060g01960 were identical but LOC104881362 was retained in the final set over VIT14s0060g01960 because it contained a longer UTR on both sides.

Nine hundred and seventy gene models out of the 5761 could be chosen for the final set only based on the HO/QO scores. The other groups were visually inspected with IGV. Many groups contained more than one true gene model which were curated and split into smaller groups, leading to in an increase of genes appearing in 2 or 3 annotations (Table 3). The sequences from the versions older than Cribi v1 (8X, or EST) that did not overlap gene models, were removed because they did not correspond to functional gene models or because there was no proof of actual expression. The final set of putative genes contained 42,414 gene models. Nearly half of them however only appeared in one single annotation, while 15,288 were constantly predicted in all 3 annotations.

**Table 3:** Correspondence between gene models within the 3 annotations. In brackets possible occurrence in CRIBI V1, VCost and Refseq respectively.

	Before manual analysis	After curation
In only one annotation (1/0/0)	**17,325**	**16,444**
In 2 annotations (1/1/0)	**6535**	7555
In 3 annotations (1/1/1)	**13,233**	15,288
Group with multiple genes (ex:2/3/1)	**5761**	**3127**
Total	**42,854**	**42,414**

A detail of the distribution of the genes models within groups is presented in Table 4. VCost was the version of annotation with the highest number of unique gene models (9831), many of these genes were very short and their existence needed to be confirmed. On the opposite, there were only 2665 Refseq specific gene models. The number of groups, for which not a single gene model from one annotation was conserved in the final set (0 or many genes in each annotation, in yellow in Table 4) was drastically reduced after curation. Among the remaining groups, two distinct cases could be distinguished. The most frequent case consisted of multiple gene models from the Refseq annotation overlapping on each other (the two other annotations algorithms did not allow overlapping). In that case, the largest gene was conserved: we only observed small gene models included in larger ones and never overlapping portions of different models. The other case consisted in genes from the families that were manually curated that were split in an annotation and not detected in the others.

**Table 4:**
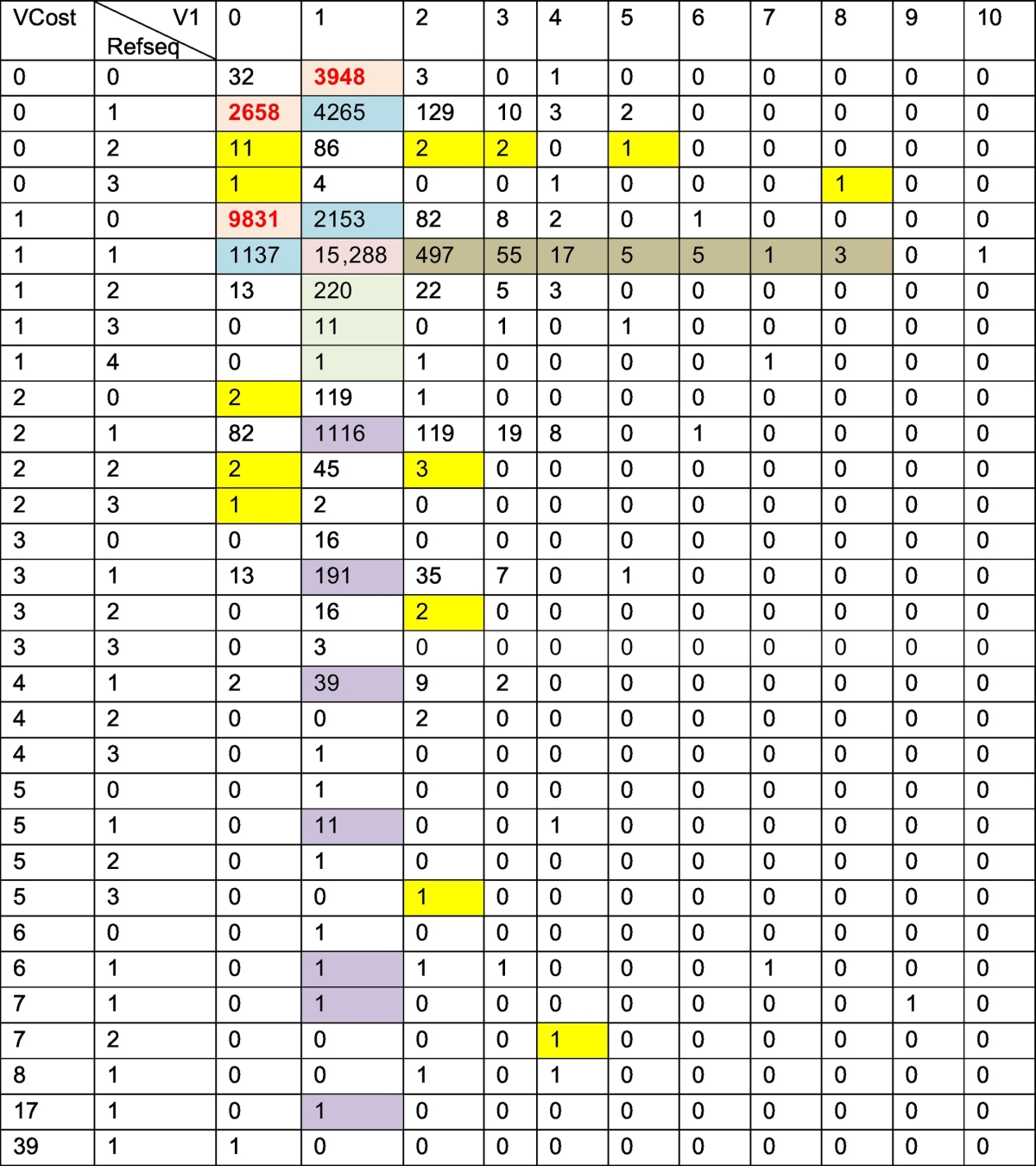
Correspondence of gene models between the three versions of automatic annotation. In bold, the gene models specific of each of them. In blue: gene models appearing in two annotations. In brown, models that were split in the V1. In purple, models that were split in the VCost. In green, models that were split in the Refseq. Yellow: models for which not a single gene model from one annotation was conserved in the final set (0 or many genes in each annotation).
